# Membrane-Fusing Vehicles for Re-Sensitizing Transporter-Mediated Multiple-Drug Resistance in Cancer

**DOI:** 10.3390/pharmaceutics16040493

**Published:** 2024-04-02

**Authors:** Sahel Vahdati, Alf Lamprecht

**Affiliations:** 1 Departments of Pharmaceutics, Institute of Pharmacy, University of Bonn, 53121 Bonn, Germany; sahelvahdati@gmail.com; 2 Pharmaceutical and Cell Biological Chemistry, Institute of Pharmacy, University of Bonn, 53121 Bonn, Germany

**Keywords:** ABC transporter, ABCG2 (BCRP), ABCB1 (P-gp), ABCC1 (MRP1), multidrug resistance (MDR), cyclosporine A, cytoplasmic delivery, membrane-fusing vehicles, fusogenic liposomes

## Abstract

Reversing the multiple drug resistance (MDR) arising from the overexpression of the efflux transporters often fails mainly due to the high toxicity or the poor water solubility of the inhibitors of these transporters. Here, we demonstrate the delivery of an inhibitor targeting three ABC transporters (ABCB1, ABCC1 and ABCG2) directly to the cell membrane using membrane-fusing vehicles (MFVs). Three different transfected MDCK II cell lines, along with parental cells, were used to investigate the inhibitory effect of cyclosporine A (CsA) in solution versus direct delivery to the cell membrane. CsA-loaded MFVs successfully reversed MDR for all three investigated efflux transporters at significantly lower concentrations compared with CsA in solution. Results showed a 15-fold decrease in the IC_50_ value for ABCB1, a 7-fold decrease for ABCC1 and an 11-fold decrease for ABCG2. We observed binding site specificity for ABCB1 and ABCG2 transporters. Lower concentrations of empty MFVs along with CsA contribute to the inhibition of Hoechst 33342 efflux. However, higher concentrations of CsA along with the high amount of MFVs activated transport via the H-binding site. This supports the conclusion that MFVs can be useful beyond their role as delivery systems and also help to elucidate differences between these transporters and their binding sites.

## 1. Introduction

ABC (ATP-binding cassette) transporters represent a diverse family of integral membrane proteins that facilitate the translocation of a broad spectrum of structurally unrelated compounds across the cell membrane. These substances encompass both endogenous substance such as lipids, ions, proteins, etc., as well as exogenous agents including xenobiotic, therapeutic drugs. These transporters utilize ATP hydrolysis to efficiently navigate through the cell membrane against the concentration gradient. Consequently, these transmembrane efflux transporters pose a significant challenge in drug delivery.

Especially in the context of intracellular delivery, these transporters play a crucial role in the failure of cancer therapies. In over 90% of the deaths arising from cancer despite chemotherapy, a phenomenon known as multiple-drug resistance (MDR) is responsible for the lack of success of treatments [1]. Among the several mechanisms involved in MDR, the overexpression of certain members of the ABC transporter family of transporters is commonly observed [1]. Interestingly, the three main protagonists involved in this scenario are also ABCB1, ABCC1 and ABCG2. The increased drug efflux leads to a lower intracellular concentration of the chemotherapeutic agents and thereby a reduced therapeutic effect on the cancer cells. Consequently, the inhibition of these transporters could potentially increase the local intracellular availability of chemotherapeutic agents and is considered a potential method to overcome cancer by re-sensitizing ABC transport-related MDR cancer cells to these drugs [2,3,4,5]. 

The administration of inhibitors targeting these transporters is the most common approach to overcoming their negative impact on drug delivery. Given that the inhibitor binding site of these transporters comprises a large and hydrophobic pocket located in the transmembrane domain (TMD), it is not surprising that the majority of developed potent inhibitors have a high molecular weight and poor water solubility [6,7,8,9]. ‘Hit compounds’ identified through computational modeling frequently demonstrate unexpected behaviors during in vitro investigations, often attributable to their poor water solubility. Consequently, a notable challenge arises in in vivo applications, stemming from the high toxicity of the inhibitors resulting from the high dose administered due to the ineffective and unselective availability at the site of action. Drug delivery systems help to increase the efficacy of the inhibitors by delivering them more efficiently to the target at lower concentrations compared with those in conventional applications in solution [10].

Several studies have explored various drug delivery mechanisms to target ABC transporters more specifically and enhance the drug solubility at the nanoscale and/or aid in their delivery to the site of action. Despite some improvement in efficacy achieved using nanoparticles or liposomes, the majority of these particles have failed to target the site of action within the transporters [11,12,13,14,15,16,17]. Since the binding site for inhibitors and substrates of these transporters is located in the transmembrane domain (TMD), it is hypothesized that inhibitors must reach their interaction site through diffusion in the membrane and/or from the cytosol. This presents a significant challenge for using nanoparticles and conventional liposomes, as they typically enter the cells via endocytosis. The delivery of the inhibitor into the cytosol using these techniques presents an opportunity to target the subcellular efflux transporters, such as those localized in the nucleus membrane and mitochondria. However, this approach bypasses the outer cell membrane, which is an advantage for delivering the substrates but not the inhibitors. In this study, we investigate the suitability of localized delivery to the TMD of the transmembrane transporters using membrane-fusing vehicles (MFV). MFVs, a subset of liposomal formulations, have recently confirmed the ability to fuse selectively with the cell membrane [18]. Given the hydrophobic nature of the majority of the inhibitors, it is anticipated that they will preferentially integrate into the MFV lipid bilayer upon formulation. Subsequent fusion with the cell membrane would facilitate direct access of the inhibitors to the TMD of the transporter proteins via the cell membrane without being released into the cytosol.

Considering the specific localization of the ABC transporters, using MFVs presents an opportunity to increase the local concentration within the transmembrane domain. This strategy would help lower the risk of inefficacy due endosomal uptake, which typically distances the inhibitor from the ABC transporters. Moreover, the inherent properties of MFVs renders them well-suited carriers for facilitating the delivery of lipophilic inhibitors into the cell membrane. Consequently, this approach addresses challenges associated with poor water solubility. The combined effect of targeted delivery and improved solubility holds promise for achieving a higher efficacy of the inhibitor at a very low concentration and avoid toxicity [18]. 

Here, we investigated the impact of MFV-based delivery on the inhibitory efficacy of cyclosporine A (CsA) as a model inhibitor. CsA is a poorly water soluble compound and is considered a first-generation inhibitor of MDR-related ABC transport, with a notably higher affinity toward ABCB1 and ABCC1 in comparison with ABCG2 [19]. For this aim, we formulated MFVs containing varying concentrations of CsA and evaluated their efficacy in reversing drug resistance toward three different MDCK II cell lines with an overexpression of the previously mentioned transporters. Our control experiments involved the use of MDCK II parental (SENS) cells lacking transporter overexpression of the membrane transporters. Additionally, we conducted substrate accumulation assays, and MDR reversal assays were performed to complement our findings. Furthermore, we explored the impact of MFVs on membrane fluidity, and its impact on the inhibitory potency of CsA.

## 2. Materials and Methods

### 2.1. Materials

1,2-dioleoyl-3-trimethylammonium-propane (DOTAP) and 1,2-dioleoyl-3-glycero-phosphatidylethanolamine (DOPE) were purchased from Avanti-polar-lipids, Inc. (Alabaster, AL, USA). The fluorescent compound 1,1′-Dioctadecyl-3,3,3′,3′-Tetramethylindocarbocyanine Perchlorate, known as DiI, and 4-(2-hydroxyethyl)-1-piperazineethanesulfonic acid (HEPES) dry powder were purchased from Merck KGaA (Darmstadt, Germany). MycoStrip^TM^ was purchased from InvivoGen (Toulouse, France). All the other chemicals were purchased from Sigma-Aldrich (Taufkirchen, Germany). For all cell-biological assays using the free drug, a stock solution of the compounds at a final concentration of 10 mM in DMSO was prepared, and, the membrane-fusing vehicle (MFV) preparation in methanol was prepared in advance and stored at 4 °C.

### 2.2. Preparation and Characterization of MFVs

The membrane-fusing vehicles containing a 1:1 ratio of DOPE to DOTAP were prepared from their stock solution in chloroform. For formulations involving lipophilic membrane staining, 100 μL from the stock solution of the fluorescent cationic lipophilic dye, DiI, at a concentration of 10 mM, was added to the lipids in chloroform. The organic solvent was removed through a slow vacuum process to create a thin layer followed by re-hydration with an appropriate volume of 20 mM HEPES buffer (pH = 7.5) to achieve a final lipid concentration of 1 mM. The assembled multilamellar vesicles (MLVs) upon hydration in HEPES were further homogenized into small unilamellar vesicles using a mini-extruder (Avanti polar lipids, USA) equipped with a 100 nm polycarbonate filter [20,21]. CsA-loaded MFVs (CsA-MFVs) were prepared in the same way, with CsA dissolved instead of DiI in chloroform.

The particle size and polydispersity index (PI) of the MFVs were measured using dynamic light scattering (DLS). The surface charge was determined by investigating the ζ-potential of the MFVs, diluted 1/40 in HEPES buffer, using Nanopartica SZ-100 (Horiba, Kyoto, Japan) at room temperature. The stability of the formulations was investigated over time by the determining these parameters periodically (up to three weeks). All measurements were taken in triplicate and are presented as mean ± SD.

Total drug loaded (DL%), which represents the proportion of drug encapsulated within a drug delivery system, and entrapment efficiency (EE%), which defines the effectiveness of a drug delivery system in encapsulating a drug within its structure (it quantifies the percentage of the total amount of drug added to the formulation that is successfully retained within the delivery system, relative to the total amount of drug initially added), were determined via high-performance liquid chromatography (HPLC). DL% is calculated using the equation [Entrapped CsA/(Entrapped CsA + MFVs)] × 100, while EE% is determined using the equation [(totalCsA − freeCsA)/totalCsA] × 100.

An amount of 1 mL of the final formulation was freeze-dried in a Steris Lyovac GT2 freeze-dryer (Mentor, OH, USA). The obtained thin layer of the dried lipid/drug mixture was then lysed in pure methanol and vortexed for 2 min. The EE% was determined indirectly via the centrifugation of the freshly prepared MFVs using Nanosep^®^ centrifugal devices (Pall, Port Washington, NY, USA) with an omega membrane MWCO of 100 KDa at 12,000× *g* for 6 min; the free drug-containing fraction was then freeze-dried and dissolved in pure MeOH. Samples were analyzed with a Waters Alliance (Waters, Milford, MA, USA) HPLC 2695, equipped with a Waters 996 photodiode array detector. CsA concentration was analyzed on the C-18 column LiChrospher 100 RP 18-5 μm EC (CS-Chromatographie, Merck, Rahway, NJ, USA). The mobile phase consisted of a mixture of acetonitrile and water buffer (75:25, *v*/*v*), and CsA was detected at a 210 nm wavelength [22].

### 2.3. Cell Culture Studies

Cell Culture. Experiments were performed on four different cell lines, all members of the Madin–Darby Canine Kidney II (MDCK II) cell line family: parenteral cells (without overexpression of transporters), MDCK II SENS; ABCG2-overexpressing cells, MDCK II BCRP; ABCB1-overexpressing cells, MDCK II MDR1; and ABCC1-overexpressing cells: MDCK II MRP1. All cell lines were received as a generous gift from Dr. A. Schinkel (The Netherlands Cancer Institute, Amsterdam, The Netherlands). The overexpressing cell lines were generated via transfection of MDCK II SENS individually with the genes encoding each of the efflux transporters. In the case of MDCK II BCRP cells, the human wild-type cDNA is C-terminally extra-linked to the cDNA of the green fluorescent protein (GFP). Cells were maintained in Dulbecco’s modified eagle medium (DMEM) supplemented with 50 µg/µL of streptomycin, 50 U/mL of penicillin G, 10% fetal bovine serum (FBS) and 2 mM L-glutamine. Cells were kept in a humidified incubator at a temperature of 37 °C with 5% CO_2_. For sub-culturing or biological evaluations, cells were harvested after reaching a confluency of 80%. For detaching the cells from the flask after removing the old medium, a mixture of 0.05% Trypsin and 0.02% EDTA was added directly to the cells and then incubated for 4 min, followed by re-suspending them gently in a fresh medium in a 50 mL falcon. After centrifugation (1200 rpm, 4 °C, 4 min), the supernatant was then removed with the help of an aspirator, and the cell pellet was either re-suspended in the fresh medium and used for sub-culturing or washed three times with PBS to remove the excess amount of medium. Mycoplasma testing was performed regularly on all cell lines using MycoStrip^TM^ used in this study to ensure the absence of mycoplasma contamination. Cell counting was performed on a CASY1 model TT supplied with a 150 µm capillary (Schaerfe System GmbH, Reutlingen, Germany).

#### 2.3.1. Confocal Laser Scanning Microscopy (CLSM)

A Nikon A1 confocal laser scanning microscope (CLSM) (Nikon, Tokyo, Japan) equipped with an Eclipse Ti-E inverted microscope (Nikon, Japan) and an LU-NV laser unit (Nikon, Japan) was used. Cells were added into the glass bottom live-imaging cell culture dishes (Ibidi Gmbh, Gräfelfing, Germany) and incubated for 24 h at 37° with 5% CO_2_. After medium removal, cells were washed 2 times with a PBS buffer, nuclei were stained using 20 µL of Hoechst 33342 (1 µM), and the cells were incubated for a further 30 min under the same conditions. After removing the excess amount of dye by washing with PBS, cells were then treated with the PBS buffer containing DiI-stained MFVs. After incubation for 15 min in the incubator, cells were washed two times with PBS, and the ability for fusion was investigated using CLSM. The images were taken using a 40× objective (oil immersion, numeric aperture 1.40) at 405 nm excitation and with blue emission for Hoechst 33342 and 543 nm and yellow emission for DiI.

The MDCK II BCRP cells were investigated using Nile-red-loaded MFVs, since there is a fluorescent overlap between GFP and DiI.

Furthermore, a three-dimensional reconstruction of the MDCK-ABCB1 cells treated with DiI-loaded MVFs was also generated. Cell preparation and microscope settings remained consistent with those previously described. Z-stack images were acquired through the entire depth of the sample, using a Z-range of 33.98 µm and a Z-Step of 0.8 µm. The top was set at 16.99 µm and the bottom was set at −16.99 µm. Nikon NIS Elements Advanced Research software (https://industry.nikon.com/de-de/produkte/industriemikroskopie/industriemikroskope/software/) (Nikon Cooperation Inc., Tokyo, Japan) was used to reconstruct three dimensional images from the Z-stack data.

#### 2.3.2. Substrate Accumulation Assay

The substrate accumulation assay is a common method to study the potency of a compound toward the transporters. The principle underlying all accumulation assays involves comparing the final concentration of a fluorescent compound that becomes actively transported through the transporter, both with and without an inhibitor. The degree of inhibition by the different drug delivery methods correlates with the measured fluorescence intensity. In our study, we utilized MDCKII parental cells, as well as MDCII BCRP (ABCG2), MDCKII MDR1 (ABCB1) and MDCKII MRP1 (ABCC1). Table 1 presents shows the list of fluorescent compounds and whether they are actively transported or not [23,24,25,26]. 

In total, 20 µL of different concentrations of the compound either in the solution or diluted from the MFV formulations was added into the 96 well-plates. Following the addition of approximately 40,000 cells in PBS per well, plates were incubated for 30 min at 37° with 5% CO_2_. Finally, the fluorescent substrate was added and incubated under equivalent conditions for two hours. After reaching the steady state, samples were studied using a Guava easyCyte8HT (Merck, Millipore, St. Louis, MO, USA) flow cytometer. Calcein and Rhodamine 123 were both excited at 488 nm, and their fluorescence was collected using a 525 ± 30 nm bandpass filter. Hoechst 33324 and Pheophorbide A were excited at 405 and 488, respectively, and their fluorescence was then collected at 448 ± 50 and 695 ± 50 nm bandpass filters [27].

#### 2.3.3. MTT Cytotoxicity Assay

For determining the intrinsic cytotoxicity of the MFVs with and without CsA, an MTT cytotoxicity assay was performed with some minor modifications. For this aim, the cells were harvested and re-suspended in the complete fresh medium at a final concentration of 200,000 cells/mL. An amount of 200 µL of the cell suspension was then seeded per well in the 96-well tissue culture plates (Sarsted, Newton, MA, USA) and stored overnight in a 37° incubator in an atmosphere of 5% CO_2_ in the humidified chamber. Afterwards, the old medium was replaced with a fresh medium containing different concentrations of the MFV formulation. A vehicle-free medium as a negative control (0% fatality), as well as a positive control of 100% fatality in the presence of 20 µL of DMSO, was also used for each cell line. Following the incubation of the cells for 24 h, 40 µL of MTT reagent was added to each well and incubated for one more hour under the previously mentioned conditions. Next, the supernatant was removed and cells were washed once with PBS, followed by the addition of 100 µL of DMSO and incubation for 1 h in the dark. The formed formazan was dissolved in the presence of DMSO, and its absorbance was determined at a wavelength of 544 nm with a background correction at 710 nm using the BMG POLARstar microplate (BMG LABTECH, Offenburg, Germany). In order to study the effect of delivering CsA via fusion into the membrane, a concentration-dependent accumulation assay was performed. For this aim, the membrane-fusing vehicles were prepared with 4 different amounts of CsA (0.25, 0.50, 0.75 and 1.00 mM) added into the formulation in the 1st step of preparation. Each cell line was individually investigated for its substrate’s accumulation in the presence of different MFV formulations [28,29]. Drug-free MFVs were tested equivalently (Supplementary Figure S1).

#### 2.3.4. Cell Toxicity of the Cytotoxic Drugs

The effect of the targeted delivery of CsA with the help of MFVs in reversing the ABC transporter-mediated MDR of the MDCKII cells with the overexpression of the transporters was investigated using an MDR reversal assay. For this purpose, 160 µL of the suspension of each cell line (MDCK II parental and ABC transporter overexpressing cells: MDCKII BCRP and MDCK II MDR1; MDCK II MRP1) was subsequently seeded in the 96-well plates, resulting in a final cell density of 20,000 cells per well, and plates were incubated overnight in an incubator containing 5% CO_2_ at 37°. After removing the old medium, cells were treated with 20 µL of fresh medium containing different amounts of MFVs. The plates were then incubated for 1 more hour in the incubator, followed by the addition of 20 µL of the cytotoxic substrate for each transporter at different concentrations. (Daunorubicin was used for cells with ABCB1 and ABCC1, and SN-38 was used for cells with ABCG2). After incubating the plates for an additional 24 h, a parallel MTT assay was performed as described in the previous chapter [28]. The treatment duration for the MTT assay was set at 24 h based on preliminary investigations that revealed significant toxicity being associated with multi-functional nanovesicles (MFVs) upon longer exposure times. Extending the treatment duration beyond 24 h resulted in substantial cell death across various concentrations of MFVs, making it challenging to observe significant differences in cell proliferation due to the severe cytotoxic effects.

### 2.4. Statistical Analysis

All analyzed data were obtained using GraphPad Prism (version 8.0, San Diago, CA, USA). Experiments were repeated at least three times on different occasions, and results are reported as mean ± SD.

## 3. Results

### 3.1. Physicochemical Characterization of MFV

Drug-free MFVs were of a size of about 100 ± 4 nm and had a zeta potential of 42 ± 5 mV. The encapsulation of CsA as well as DiI did not significantly affect the size and surface potential of the MFVs (Table 2). CsA entrapment efficiency for different formulations was in the range of 74 to 98%, while drug load was between 9 and 23% for different formulations (Table 2). Storage up to 3 weeks at under 4 °C showed a slight but not significant change in size but no change in drug load.

### 3.2. Imaging of Membrane-Fusing Vehicles

**Figure 1:**
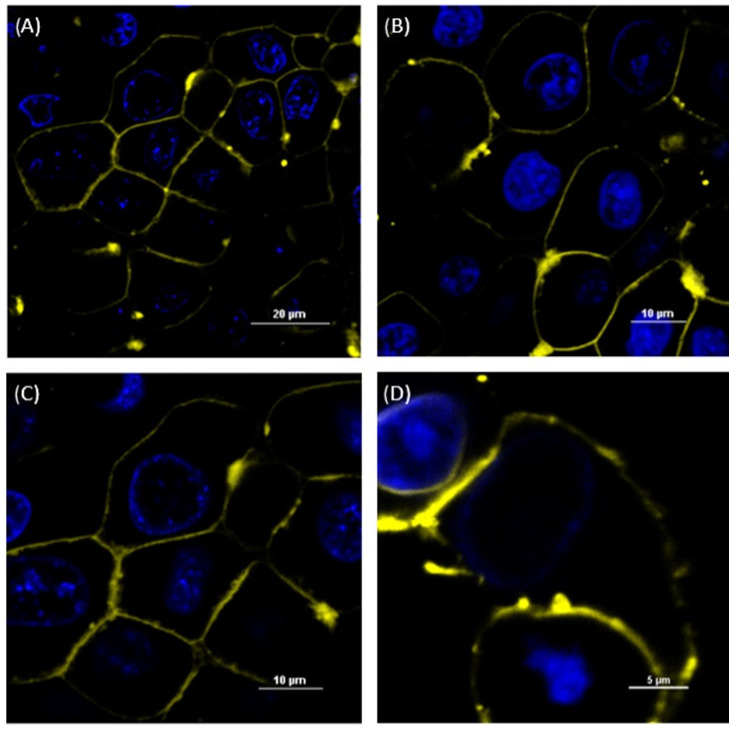
Confocal laser scanning microscope (CLSM) images of MDCK II SENS (**A**), MDCK II ABCB1 (**B**) and MDCK II ABCC1 (**C**) incubated with the DiI-stained MFVs and MDCK II BCRP (**D**) incubated with Nile red-stained MFVs for 15 min. The nucleus was stained with Hoechst 33342. The outer membrane was stained by adding the MFVs containing DiI after 15 min of incubation, indicating the fusion of DiI-containing MFVs into the cell membrane. The scale bar is 20 µm for (**A**), 10 µm for (**B**,**C**) and 5 µM for (**D**). We observed the agglomeration of some of the MFVs after their addition to PBS.

**Figure 2:**
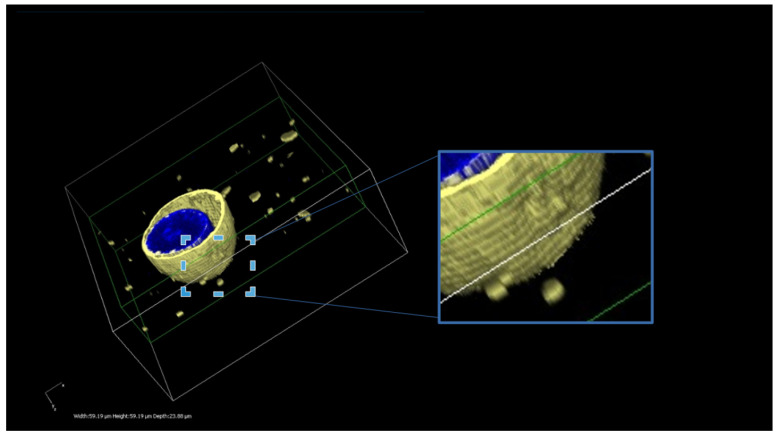
A three-dimensional reconstruction of the MDCK-ABCB1 cells treated with DiI-loaded MVFs. The presented image illustrates the successful delivery of DiI using MFVs. The uniform and homogeneous incorporation of DiI into the cell membrane is visible. Furthermore, this live 3D image offers an insightful glimpse into the interaction between the MFVs and the cell membrane. The scale bars in 3 dimensions are width 59.19 µm, height 59.19 µm and depth 23.88 µm.

Following the incubation of the cells with DiI or Nile-red-loaded MFVs, outer-cell membrane staining was detectable via CLSM (Figure 1 and Figure 2). These findings confirmed the fusion capability of these vehicles, while we observed almost no endosomal uptake during the incubation period. The general pattern remained similar for all investigated cell lines (MDCK II parental, ABCB1, ABCC1 and ABCG2) regardless of the existence and type of transporter overexpression.

### 3.3. Analysis of In Vitro Experiment Results: Key Observations

#### 3.3.1. Substrate Accumulation Assay

**Figure 3:**
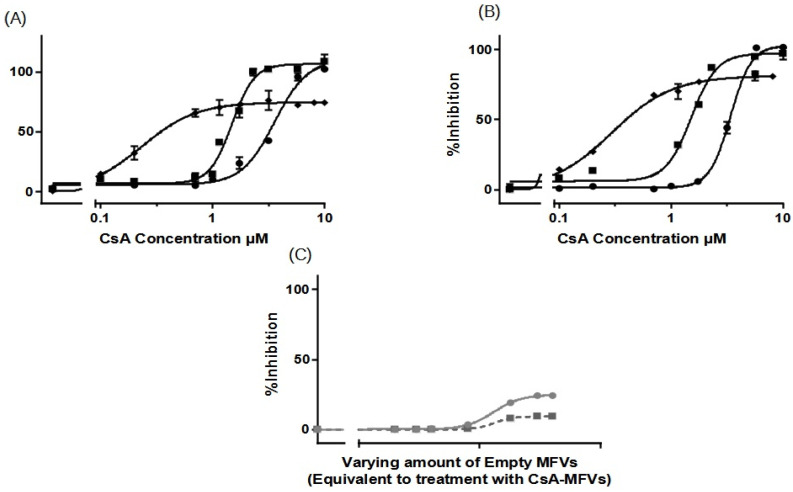
Dose–response curves, for the inhibition of ABCB1 using CsA in solution (●), compared with CsA delivered via MFVs (◆), or the co-treatment of cells with drug-free vehicles alongside CsA in solution (■), were generated using two different substrate accumulation assays: (**A**) the Hoechst 33342 assay and (**B**) Rhodamine 123 accumulation assay. Similar patterns were evident in both substrate accumulation assays. Notably, there was a pronounced leftward shift in the dose–response curve when delivering CsA via MFVs compared to with when delivering CsA in solution. The utilization of CsA loaded in MFVs yielded a 15-fold decrease in IC_50_ in the Hoechst 33342 assay (**A**) and an 11-fold decrease in IC_50_ in the Rhodamine 123 assay (**B**). Additionally, the co-administration of drug-free vehicles with CsA in solution induced a leftward shift. Moreover, a notable finding was observed regarding the I_max_ values obtained in both accumulation assays. Treatment with CsA in solution, either administered alone or in combination with drug-free MFVs, resulted in I_max_ values approximately 20–30% higher than those observed in cells treated with CsA-loaded MFVs. A negative control of drug-free MFVs (**C**) was performed, employing the same vehicle concentrations as those of the CsA MFVs to demonstrate the effect of the lipid–protein interaction on the transporter function (shown as a gray line for the Hoechst 33342 assay and a gray dotted line for the Rhodamine 123 assay). Results were normalized by setting the fluorescent intensity achieved from MDCK II SENS to 100% and that from the untreated MDCK II MDR1 cells to 0%.

**Figure 4:**
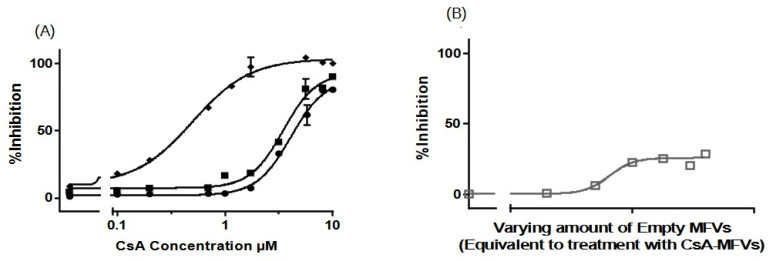
Dose–response curves, for the inhibition of ABCC1 using CsA in solution (●) in comparison with CsA delivered via MFVs (◆) or via the co-treatment of cells with drug-free vehicles in conjunction with CsA in solution (■), were investigated in a flow cytometry Calcein assay (**A**). Significant leftward shift observed in dose–response curves for CsA-MFVs compared with free CsA alone or in combination with empty carriers. A negative control of drug-free vehicles (**B**) at similar concentrations of MFVs loaded with CsA was performed to show the effect of the lipids on the inhibition of the transporter (gray line together with squares (□) for the measurement points). Figure 4A illustrates a notable leftward shift for the cells treated with CsA-MVS (◆), indicating enhanced potency. In this context, the IC_50_ values achieved were almost 8 times lower than those observed with free CsA. Co-treatment with CsA in solution alongside drug-free MFVs resulted in a 1.2-fold lower IC_50_ value. However, for ABCC1, the I_max_ value did not exhibit significant differences across the treatments. Results were normalized by setting the fluorescent intensity achieved from parental cells to 100% and the untreated overexpressing cell substrate uptake to 0%.

**Figure 5:**
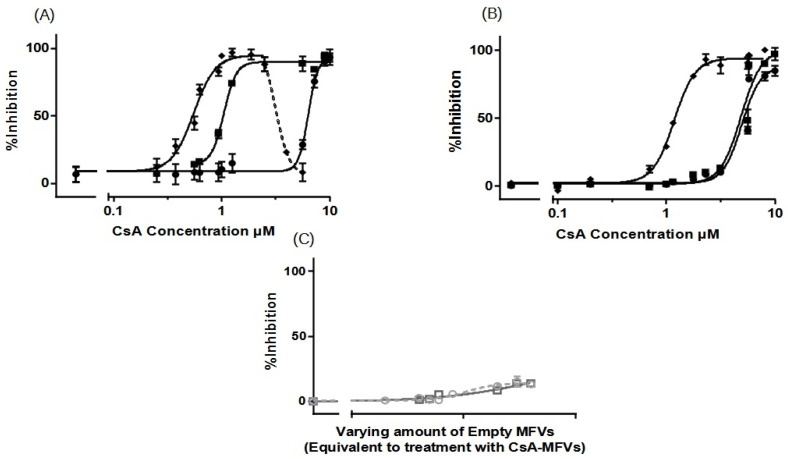
Dose–response curves, for the inhibition of ABCG2 using CsA in solution (●) in comparison with CsA delivered via MFVs (◆) or the co-treatment of cells with drug-free vehicles in conjunction with CsA in solution (■), were investigated using (**A**) Hoechst 33342 and (**B**) Pheophorbide A. The results of the Hoechst 33342 assay demonstrate a significant shift in the inhibitory efficacy of CsA towards ABCG2 when delivered via MFVs or in the presence of empty MFVs. LogIC_50_ values from the Hoechst 33342 assay were as follows: CsA in solution, 6.361 µM; CsA + drug-free MFVs, 1.018 µM; and drug-loaded MFVs, 0.5514 µM (**A**). In the Pheophorbide A assay (**B**), delivering CsA via MFVs also significantly enhanced its inhibitory effect on ABCG2. Comparing the inhibitory effects of CsA at a final concentration of 1.7 µM across various treatments highlights the enhanced delivery efficacy of MFVs for CsA. Cells treated with CsA in solution exhibited 5.0 ± 0.4% inhibition, while co-treatment with 0.8 µM empty MFVs resulted in 7.89 ± 0.4% inhibition. In contrast, delivering CsA via MFVs significantly increased inhibition, with the active transport of Pheophorbide A via ABCG2 reaching 83.8 ± 4.2% (**B**). A negative control of drug-free vehicles at similar concentrations of MFVs loaded with CsA, was performed to show the effect of the vehicles on the inhibition of the transporter (gray dotted line with circles for the Hoechst 33342 assay and darker gray line with squares for the Pheophorbide A assay) (**C**). Results were normalized by setting the fluorescent intensity achieved from parental cells to 100% and the untreated overexpressing cell substrate uptake to 0%.

**Figure 6:**
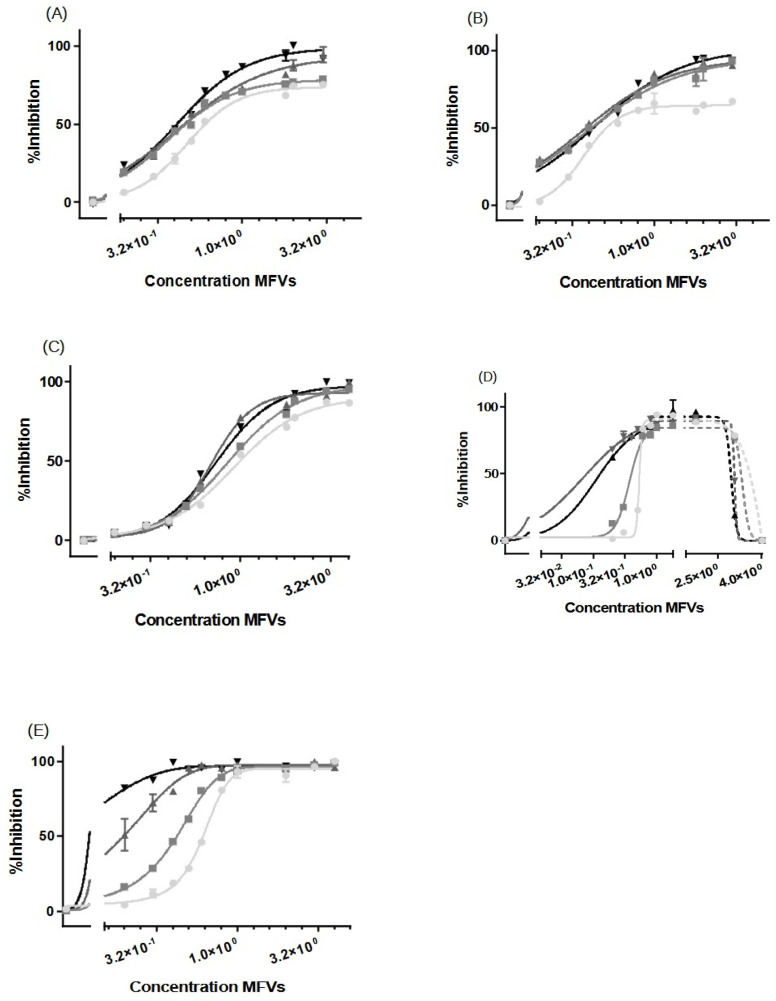
Increasing concentrations of MFVs (0.02–4 µM: shown as a power of 10 in the graphs) plotted against the % inhibition of resistant cell lines. A higher gray color intensity indicates a higher amount of CsA in the formulation (● = 0.25 mM, ■ = 0.50 mM, ▲ = 0.75 mM and ▼ = 1.00 mM). Assays were performed on the ABCB1-overexpressing MDCK II MDR1 cells in the (**A**) Hoechst 33342 accumulation assay and (**B**) Rhodamine 123 accumulation assay. ABCC1-overexpressing MDCKII MRP1 cells used in the Calcein accumulation assay (**C**). ABCG2-expressing MDCKII BCRP cells used in a (**D**) Hoechst 33342 assay and (**E**) Pheophorbide A assay. For ABCB1, regardless of the substrate used, the formulations with a higher amount of CsA achieved about a 30% higher I_max_ value in comparison with formulations with lower amounts of CsA; this way, total inhibition was achieved. A slight leftward shift was also observed when using CsA MFVs with higher concentrations of CsA. The pattern exhibited by ABCC1 cells closely mirrored that observed for ABCB1. For cells with an overexpression of ABCG2, the increased amount of CsA at lower concentrations of MFVs showed a strong inhibitory effect in the Hoechst 33342 and Pheophorbide A accumulation assay. However, at higher concentrations of MFVs, the amount of CsA delivered led to a disadvantage to the inhibitory effect of CsA toward ABCG2 in Hoechst 33342 accumulations. Results were normalized to the I_max_ value achieved using SENS cells as 100% and using untreated resistant cells as 0%.

The inhibitory activity of CsA delivered using MFVs was compared with that of CsA added in solution. Notably, a pronounced leftward shift in the dose–response curve is evident using CsA MFVs (Figure 3, Figure 4, Figure 5 and Figure 6). These findings indicate a substantial decrease in the minimum concentration of the drug needed to achieve 50% of inhibition. The delivery of CsA using MFVs toward ABCB1 led to a 15-fold reduction in the IC_50_ value, as determined by the lower IC_50_ in the Hoechst 33342 accumulation assay (Figure 3A). IC_50_ values achieved from the Hoechst 33342 assay are as follows: for CsA in the solution, 3.582 µM; for CsA together with 0.8 µM drug-free MFVs: 1.492 µM; and for CsA MFVs, 0.2331 µM. At a final concentration of approximately 1.7 µM, CsA in solution achieved 24 ± 4% inhibitions of the transporters in the Hoechst 33342 accumulation assay. In contrast, the concurrent treatment of cells with the drug-free MFVs and CsA increased inhibition to 67 ± 5%, while CsA-loaded MFVs demonstrated the highest inhibition at 73 ± 7% under similar concentrations.

Results obtained from Rhodamine 123 accumulation assay were in line with the findings from the Hoechst 33342 accumulation assay. Here, IC_50_ values were as follows: CsA in the solution: 3.383 µM; CsA together with 0.8 µM drug-free MFVs: 1.515 µM; and CsA MFVs, 0.2965 µM (Figure 3B). The inhibitory potency of CsA in solution (at 1.7 µM) was about 8 ± 2%, while the co-treatment of CsA and 0.8 µM MFVs increased the inhibitory potency of the drug up to 62 ± 2%. The delivery of the same amount via CsA MFVs showed almost an 77 ± 1% inhibition of the ABCB1 transporters. Cells treated with CsA alone or together with drug-free MFVs resulted in approximately 20–30% higher I_max_ values in comparison to those of the cells treated with CsA MFVs, for both Hoechst 33324 and Rhodamine 123 accumulation assays. As a negative control, the effect of MFVs on the transporter at the equivalent final concentrations used for CsA MFVs showed no significant results in both assay methods for ABCB1 (Figure 3C).

We extended our studies to investigate the effect of MFVs on inhibiting the transport of substrates through ABCC1 transport (MRP1) in a Calcein accumulation assay (Figure 4A). The dose–response curves obtained from CsA-MFVs on ABCC1 show a similar pattern to those found with ABCB1: a shift to the left (Figure 4A). In this case, the IC_50_ values achieved were almost eight times lower than those for the free CsA, while the co-treatment of drug-free MFVs together with CsA in the solution led to a 1.2-fold lower IC_50_ (LogIC_50_ values achieved from the Calcein assay are as follows: for CsA in solution: 4.08 µM; for CsA + drug-free MFVs: 3.47 µM; and for drug-loaded MFVs: 0.500 µM). Likewise, the inhibitory efficacy of CsA at its final concentration of 1.7 µM for CsA in solution is 7.3 ± 0.5%, where the co-treatment of drug-free MFVs increases the inhibitory percentage of 1.7 µM CsA up to 18.4 ± 0.3% and the same amount of the drug delivered inside MFVs inhibits almost 14 times more potently (97 ± 7%). In case of ABCC1, the I_max_ value did not differ significantly between the treatments. As a negative control, the effect of MFVs on ABCC1 at equivalent final concentrations used for CsA MFVs showed no significant results in both assay methods for ABCB1 (Figure 4B).

The further investigation of CsA delivery via MFVs targeting ABCG2 also revealed a notable decrease in the minimum CsA concentration required to achieve a 50% inhibitory effect compared with that of CsA in solution. The LogIC_50_ values from the Hoechst 33342 assay were as follows: CsA in solution, 6.361 µM; CsA + drug-free MFVs, 1.018 µM; and drug-loaded MFVs: 0.5514 µM (Figure 5A).

Observing the re-sensitization of the cells overexpressing the ABCG2 transporter at a final concentration of 1.7 µM CsA in the Hoechst 33342 accumulation assay showed that the inhibitory efficiency of CsA in the solution was 11.8 ± 3.2%, where the co-treatment of the same amount of CsA in the solution together with drug-free vehicles at a concentration of 0.8 µM re-sensitized almost fully (94.7 ± 2.2%), while MFVs loaded with the same amount of CsA helped reverse the resistance (95.2 ± 3.8%).

The results from the Pheophorbide A accumulation assay (Figure 5B) showed that the IC_50_ of CsA in solution was 5.41 µM, where the presence of drug-free MFVs helped it reach a value of 4.60 µM, and for CsA delivered via MFVs, the IC_50_ is 1.22 µM.

The comparison of the inhibitory effect of CsA at its final concentration of 1.7 µM for different treatments highlights the effect of MFVs on delivering CsA more clearly. The cells treated with CsA in the solution reached 5.0 ± 0.4% inhibition, while the presence of 0.8 µM empty MFVs showed an inhibition of 7.89 ± 0.4%. Delivering CsA via MFVs inhibits the active transport of Pheophorbide A via ABCG2 up to 83.8 ± 4.2%.

In the dose–response curve obtained from Hoechst 33342 accumulation assay where ABCG2 was investigated (Figure 5A) after reaching the I_max_ plateau indicating the saturation of the transport protein, the addition of a higher amount of the CsA MFVs vehicles led to a decrease in the fluorescent intensity. In other words, it appears that the transporter actively transported Hoechst 33342 at higher concentrations of CsA MFVs. A similar pattern was observed at higher concentrations of empty MFVs together with the higher concentration of CsA (Supplementary Figure S3).

Interestingly, the co-treatment of drug-free MFVs at their different concentrations together with several CsA concentrations did not show any changes in the uptake of Pheophorbide A (Supplementary Figure S4). Unlike that for ABCB1, the I_max_ had no significant differences for all treatments.

The impact of empty MFVs on the transporter, at equivalent final concentrations utilized for CsA-MFVs, did not yield any outcomes in either of the assay methods targeting ABCG2 (Figure 5C).

#### 3.3.2. Concentration-Dependent Drug Delivery Using MFVs

As previously demonstrated in Figure 5A, the dose–response curve obtained from ABCG2 exhibited a decrease in the accumulation of Hoechst 33342 at a final lipid concentration higher than 2 µM together with a high concentration of CsA being loaded. Initially, the underlying cause of the activation of Hoechst 33342 transport via ABCG2 at higher concentrations of CsA MFVs was unclear. To explore this further, we investigated whether or not CsA exhibits distinct interactions with the binding pockets of ABCG2 when delivered at elevated concentrations directly through the membrane. Four distinct formulations were prepared and utilized in the substrate accumulation assay (Table 2).

For ABCB1 and ABCC1 (Figure 6A–C), we observed a concentration-dependent inhibitory effect, with transporter saturation occurring at higher drug concentrations. Notably, a slight leftward shift was observed for higher concentrations of CsA MFVs. Specifically, at a vehicle concentration of 0.3 µM, formulations with higher CsA concentrations achieved more than twice the inhibition compared with that of formulations with 0.25 mM CsA. Additionally, in both the Hoechst 33342 (Figure 6A) and Rhodamine 123 assays (Figure 6B), formulations with higher CsA concentrations achieved complete inhibition, whereas the 0.25 mM formulation only reached an I_max_ of approximately 70–75%. In terms of the ABCC1 (Figure 6C), the increased amount of CsA loaded in the vehicles shows a similar pattern of a better IC_50_ value for formulations with higher amounts of CsA; however, the difference is less pronounced.

The increasing inhibitory effect of higher amounts of CsA delivered using lower concentrations of MFVs was more pronounced toward ABCG2 (Figure 6D,E). Treating cells with 0.3 µM of MFVs loaded with 0.25 mM CsA led to around 6% inhibition, whereas the formulation with 0.5 mM CsA reached around 25% inhibition and formulations with 0.75 and 1.0 mM of CsA reached ≈ 77% inhibition of the total ABCG2 transporters in the cells with overexpression (Figure 6D). Interestingly, at higher concentrations of MFVs, higher amount of drug delivered via the membrane also led to the activation of the transporter and increased the efflux of Hoechst 33342. However, this was not found with Pheophorbide A (Figure 6E).

**Figure 7:**
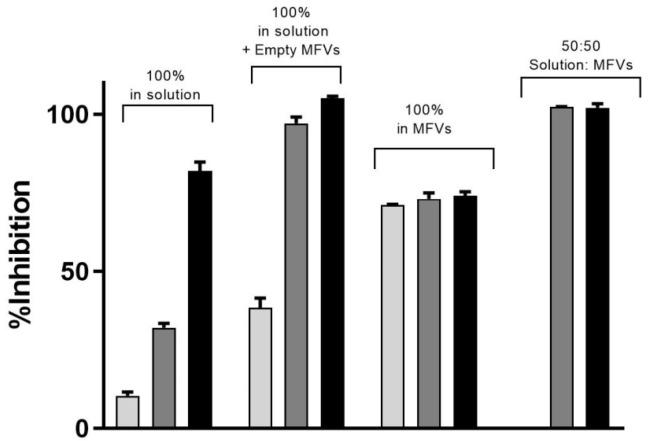
Three different concentrations of CsA’s inhibitory effect toward the efflux of Hoechst 33342 via ABCB1 shows that despite the increasing efficacy of CsA at its very low concentration, the maximum inhibition achieved remained same despite the increasing concentrations of CsA (light grey: 1.25 µM; dark grey: 2.5 µM; black: 5 µM). To reach a 100% inhibitory effect on ABCB1, it is necessary to deliver the CsA intracellularly as well. The benefit of using MFVs for CsA inhibitory efficacy is clear in all four combinations. Notably, the presence of CsA in the solution was crucial for achieving the maximum inhibitory effect (I_max_) of 100%.

In Table 2, it is observed that higher concentrations of CsA added to the formulation resulted in lower encapsulation efficiency (EE%). To investigate whether or not the observation of a higher I_max_ achieved for cells with ABCB1 when using higher concentrations of CsA MFVs (Figure 6A,B) is related to the delivery pathway of inhibitor, we conducted an investigation by combining CsA (0.25 mM)-loaded MFVs with the same amount of CsA in solution for the Hoechst 33342 accumulation assay targeting ABCB1. This comparison of four different treatment combinations at a final concentration of 2.5 µM of CsA provided valuable insights into the inhibition of ABCB1. Specifically, we compared 2.5 µM CsA in the solution alone, 2.5 µM CsA in the solution together with drug-free MFVs, and MFVs loaded with 2.5 µM CsA, with 1.25 µM CsA in the solution together with 1.25 µM CsA loaded in MFVs. The presence of MFVs alone or loaded with CsA led to a higher efficacy (lower IC_50_) of CsA compared with that of cells treated only with CsA added to the solution. However, it is interesting to note that the presence of CsA in the solution was necessary to reach the maximum inhibitory effect (I_max_) of 100% (Figure 7).

**Figure 8:**
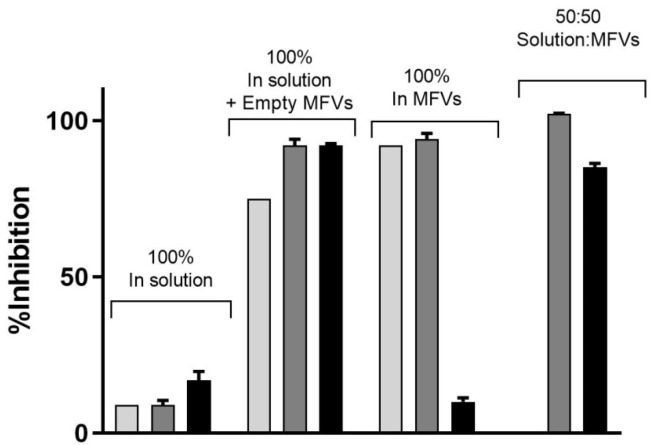
The inhibitory effect of CsA at its three different concentrations on the efflux of Hoechst 33342 via ABCG2 (light gray: 1.25 µM; dark gray: 2.5 µM; black: 5 µM). The presence of empty MFVs effectively blocks the transport of Hoechst 33342 for all investigated concentrations. However, delivering CsA at its highest concentration (5 µM) via MFVs fully activates the transport of Hoechst 33342. Conversely, delivering lower amounts of CsA may facilitate more efficient binding to the inhibitory site of the transporter, resulting in the inhibition of its activity.

Expanding on the results depicted in Figure 6D, our analysis of ABCG2 inhibition in the substrate accumulation assays using MFVs loaded with varying CsA concentrations revealed intriguing trends, which were particularly noticeable at lower CsA MFV concentrations. To delve deeper into these findings, we explored combinations of CsA MFVs with CsA solutions. Surprisingly, while MFVs loaded with 5 µM of CsA seemed to activate ABCG2 transport, delivering 2.5 µM via MFVs in conjunction with an additional 2.5 µM of CsA solution demonstrated a comparatively higher inhibitory effect (Figure 8).

#### 3.3.3. Cell Toxicity of the Cytotoxic Drugs

**Figure 9:**
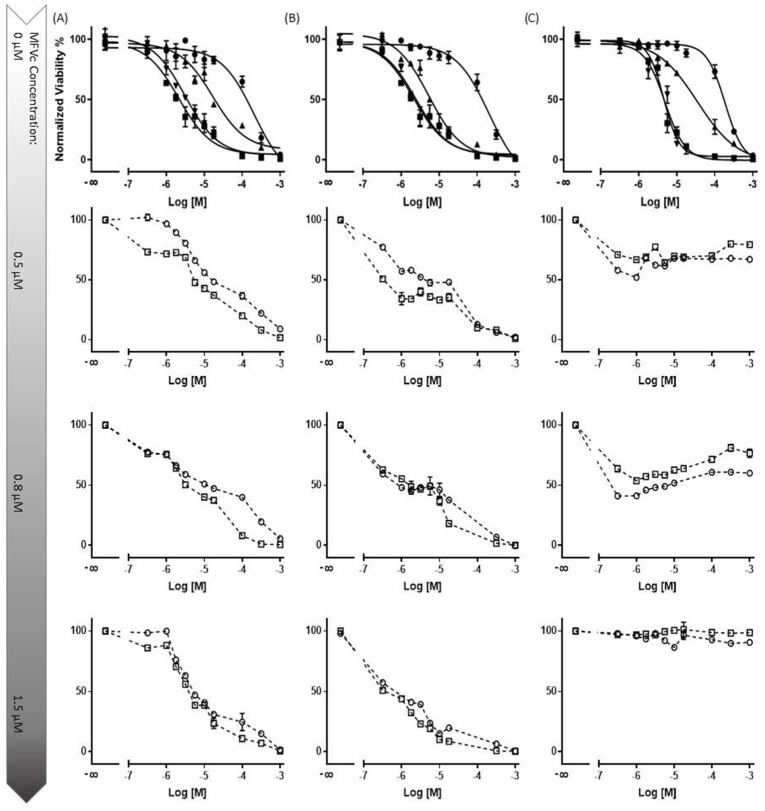
The concentration-dependent multidrug resistance (MDR) reversal assay conducted using an MTT-based viability assay across all four cell lines. The MDCK II parental (■) cell line served as the control cell line for each dataset. Dose–response curves were generated for cells with an overexpression of MDR transporters without treatment (●), and with 5 µM (▲) and 10 µM (▼) of CsA, alongside varying concentrations of cytotoxic substrates. Each column represents a specific cell line and transporter: MDCK II MDR1 (Daunorubicin) (Column (**A**)), MRP1 (Daunorubicin) (Column (**B**)), and ABCG2 (SN-38) (Column (**C**)). In total, three different final MFV concentrations (0.5, 0.8 and 1.5 µM) of 2 different formulations (○: CsA-MFV/0.5 mM and ☐: CsA-MFV/1.0 mM CsA-load) were investigated. In the first row, it is evident that the presence of the inhibitor at a final concentration of 10 µM effectively reverses the resistance of MDCK II cells overexpressing all three transporters. At a concentration of 5 µM, CsA induces intermediate re-sensitization for ABCB1 and demonstrates relatively lower efficacy for ABCG2. In row two, for dataset A (ABCB1-overexpressing cells), the utilization of 1.2 µM CsA via MFVs demonstrates comparable inhibitory potency to that of CsA in solution at a final concentration of 5 µM. However, at a final concentration of 0.5 µM, MFVs delivering 1.2 µM CsA (○) exhibit a slightly lower impact, particularly at lower cytotoxic drug concentrations, compared with MFVs delivering 2.4 µM CsA (☐). Transporter saturation occurs at lower concentrations due to the effective delivery of CsA by MFVs for both ABCB1 and ABCC1. In the case of ABCG2 cells (dataset (**C**)), lower concentrations of MFVs containing varying amounts of CsA prove to be slightly more effective. For instance, utilizing 1.2 µM CsA via MFVs results in over 60% toxicity at approximately 0.3 µM SN-38, whereas higher concentrations of MFVs lead to complete resistance to SN-38.

We conducted a concentration-dependent multidrug resistance (MDR) reversal assay using an MTT-based viability assay across all four cell lines (Figure 9). For each dataset, MDCK II parental cells were utilized as the control (marked as ■). Dose–response curves were generated to assess the impact of treatment with varying concentrations of CsA (5 µM and 10 µM) alongside different cytotoxic substrates (for the first row). Each column in the dataset corresponds to a specific cell line and transporter: MDCK II MDR1 (Daunorubicin) (Column A), MRP1 (Daunorubicin) (Column B), and ABCG2 (SN-38) (Column C). In the first row, the data demonstrate that the presence of the inhibitor at a final concentration of 10 µM effectively reverses the resistance of MDCK II cells overexpressing all three transporters. At a concentration of 5 µM, CsA induces intermediate re-sensitization for ABCB1 and exhibits comparatively lower efficacy for ABCG2.

Subsequent rows (2 to 4) represent the evaluation of three final concentrations of MFVs (0.5, 0.8, and 1.5 µM) derived from two different formulations (○: CsA-MFV/0.5 mM and ☐: CsA-MFV/1.0 mM CsA-load).

In row two, for dataset A (ABCB1-overexpressing cells), the utilization of 1.2 µM CsA via MFVs demonstrated comparable inhibitory potency to that of CsA in solution at a final concentration of 5 µM. However, at a final concentration of 0.5 µM, MFVs delivering 1.2 µM CsA exhibited a slightly lower impact, particularly at lower cytotoxic drug concentrations, compared to MFVs delivering 2.4 µM CsA. Transporter saturation occurred at lower concentrations, due to the effective delivery of CsA by MFVs for both ABCB1 and ABCC1. In the case of ABCG2 cells (dataset C), lower concentrations of MFVs containing varying amounts of CsA were slightly more effective. For example, utilizing 1.2 µM CsA via MFVs resulted in over 60% toxicity at approximately 0.3 µM SN-38, whereas higher concentrations of MFVs led to complete resistance to SN-38. These observations were consistent with the findings from the Hoechst 33342 accumulation assay, highlighting the significance of optimizing the ratio of CsA to MFVs for the efficient reversal of multidrug resistance (MDR).

## 4. Discussion

The delivery of CsA via MFVs into the TMD, bypassing endocytotic pathways, significantly reduces the minimum concentration required to re-sensitize the resistant cancer cells compared with that required when adding CsA to the solution. This approach resulted in a substantial decrease in the IC_50_ value: a more than 15-fold reduction for ABCB1, a 7-fold reduction for ABCC1 and a 11-fold reduction in the case of ABCG2. These findings confirm the effectiveness of delivering hydrophobic compounds directly into the cell membrane using MFVs. Through this, MFVs address two key challenges simultaneously: they enable the selective delivery of lipophilic inhibitors to the TMD and prevent drug loss into lysosomes by avoiding endocytosis.

**Figure 10:**
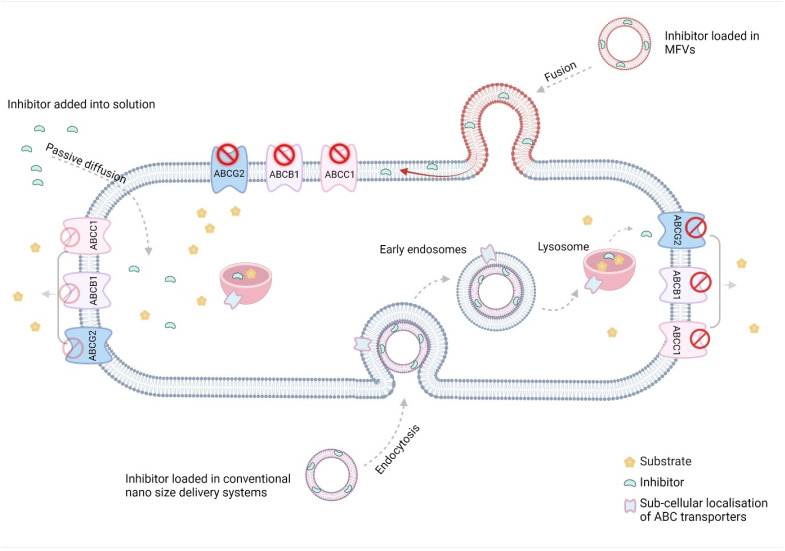
The low water solubility of efflux transporter inhibitors, combined with the stiffness of resistance cell membranes, poses challenges for the passive diffusion of these inhibitors’ inhibitory effects. While most conventional nano-sized delivery systems address the solubility issue, they often fail to tackle a primary obstacle to poor bioavailability: the inactivation of drugs taken up via endocytosis in the endo/lysosomes. The unique characteristics of MFVs present an excellent opportunity to deliver lipophilic inhibitors of ABC transporters directly to their site of action in the transmembrane domain (TMD) of the cell membrane (The figure was created using BioRender.com. Acceded on 15 April 2022).

Remarkably, the inhibition of ABCB1 up to 70% occurred at very low CsA concentrations. However, our data indicate that to achieve full inhibition (for the remaining 30%, approximately), the intracellular delivery of CsA may be necessary. This observation could be attributed to the subcellular expression of ABCB1, particularly its presence on lysosomes, which assists in neutralizing substrates of these transporters within the cells. Currently, it is suspected that ABCB1 has two substrate-binding sites; Hoechst 33342 binds to the H-binding site, while Rhodamine 123 interacts with the R-binding site [30]. In order to elucidate the potential site-selectivity of the MFV-based delivery of the inhibitor via the cell membrane to the transmembrane domain of the transporters, we investigated the accumulation of both fluorescent substrates. While the improvement in the inhibitory efficacy of CsA carried in MFVs shows a similar pattern regardless of the transporter and the analytical method, the results of simultaneous treatments of the drug-free MFVs together with CsAthe solution, has a different pattern for Hoechst 33342 accumulation depending on the MFV concentration. Lower concentrations of these vehicles induce the inhibitory effect of CsA at its lower concentrations. This could be explained via potential changes in membrane fluidity, which in turns defines membrane permeability as well as the intramembrane transit of the lipophilic inhibitors [15]. Cholesterol is an essential membrane component that determines membrane rigidity. A higher cholesterol amount in the cell membrane induces mechanical stiffness and contributes to drug resistance together with the overexpression of the ABC transporters [15]. The fusion of the MFVs at their lower concentrations helps alter membrane fluidity and eases the intramembrane transit of the inhibitors. On the other hand, increased permeability leads to higher passive diffusion of the substrates across the membrane [15]. The higher concentration of the MFVs together with high concentrations of CsA in solution led to a disadvantage to the inhibitory efficacy of CsA toward the efflux of Hoechst 33342 via both ABCB1 and ABCG2 (Figure 10).

ABCG2 stands out from ABCB1 and ABCC1 due to differences in its substrate specificity, tissue distribution, and response to various modulators. While CsA shows weak inhibitory effects on ABCG2, its influence on transporter activity differs from that on ABCB1 and ABCC1. Furthermore, the uneven distribution of lipids in the membrane, particularly the prevalence of cholesterol-rich regions where ABC transporters reside, is acknowledged. Notably, the crystal structure of ABCG2 reveals the presence of two cholesterol molecules bound within the multidrug pocket located in the transmembrane domain [31,32]. It is suggested that lipids such as cholesterol can act as reservoirs for ABC transporter substrates during the phase transition of the transporter, and through that, participate in active efflux. The fusion of MFVs might promote the relocation of cholesterol molecules into the transmembrane domain, potentially enhancing the activity of these transporters. This suggests the potential site-selective modification of ABCB1 and ABCG2 transporters exposed to MFVs. However, confirmatory control experiments involving MFVs containing cholesterol were unsuccessful, primarily due to significant alterations in the membrane properties of MFVs, resulting in the complete loss of their fusion capability.

In our study, we observed a key behavioral divergence among the transporters when MFVs with differing CsA concentrations were introduced. Lower concentrations of MFVs led to a heightened inhibition of ABCG2, particularly with high-CsA MFVs. Conversely, higher concentrations of vehicles activated ABCG2, especially when laden with increased CsA levels. This phenomenon likely stems from architectural disparities between ABCG2 and ABCB1/ABCC1. ABCG2 has a narrow slip that is accessible from the membrane and the cytosolic site. This narrow slit is where the binding pockets are located [3]. The positioning of the nucleotide-binding domains (NBDs) is notably different in ABCG2 compared with that in ABCB1 and ABCC1. In ABCG2, the NBDs are closer to the membrane, facilitated by a shorter linker in the cytoplasm. In contrast, ABCB1 and ABCC1 exhibit a distinct separation between the NBDs and the cell membrane, characterized by a clear tail. Additionally, during the transition phases of the inhibitor, an occlusion of the substrate-binding site occurs in ABCB1, but not in ABCG2 [33]. 

Previous research has demonstrated that compounds can reach their site of action either through diffusion across the cell membrane or via direct access from the cytosol [3]. However, it has remained unclear whether the cytosolic or membrane pathway has different effects. Recently, the concept of a substrate–inhibitor continuum, particularly regarding poor substrates of ABCG2, has been proposed. This concept suggests that the behavior of a compound depends on its concentration or orientation within the transporter cavity [3]. The research findings demonstrated that at elevated concentrations, two tariquidar molecules obstruct the cavity by adopting a downward L shape (Γ). This suggests that at lower concentrations, the compound traverses the cavity as a single molecule. However, at higher concentrations, two compound molecules form a Γ shape, effectively blocking the cavity and acting as inhibitors. Our findings shed light on the mechanisms underlying these observations and underscore the significance of the delivery pathway for ABCG2 inhibitors. We propose that the concentration-dependent behavior stems from the direction in which the compound enters the membrane cavity. Specifically, to form the Γ shape, one molecule must enter from the membrane side while the second molecule should originate from the cytosol. When delivering weak inhibitors (such as CsA) of ABCG2 using MFVs, the inhibitor only passes through the membrane and misses interaction with the binding site when delivered from the cytosol direction. Therefore, it is crucial to adjust the drug-to-lipid ratio to achieve the maximum inhibition of ABCG2.

## 5. Conclusions

The results indicate the feasibility of locally delivering ABC transporter inhibitors via their direct insertion into the cell membrane. The fusogenic properties of MFVs present an efficient approach for inhibiting efflux transporters through direct membrane delivery. Beyond the initial rationale for enhancing inhibitor delivery, our findings suggest promising prospects for future binding site-selective delivery strategies. Moreover, our study underscores the utility of these vehicles in discerning between different pumps and their mechanisms of action in membrane transport, despite their shared characteristics.

## Figures and Tables

**Table 1:** List of the fluorescent substrates of the MDR-related ABC transporters.

Transporter vs. Substrate	Hoechst 33324	Rhodamine 123	Pheophorbide A	Calcein
ABCB1		+		+
ABCG2	+	−	+	−
ABCC1	−	+	−	+

**Table 2:** The different particle sizes (z-average), zeta potentials and CsA drug loads as well as entrapment efficiencies for the different MFVs formulations loaded with 4 different amounts of CsA. (n = 3; mean ± SD).

MFVs	Particle Size (nm)	Zeta Potential (mV)	Drug Load (%)	Entrapment Efficiency (%)
Drug Free	110 ± 4	42 ± 5	-	-
With DiI	112 ± 7	40 ± 3	-	-
With Nile-red	127 ± 0	47 ± 6	-	-
With CsA (0.25 mM)	104 ± 0	41 ± 3	9 ± 1%	98 ± 1%
With CsA (0.50 mM)	103 ± 0	41 ± 0	16 ± 2%	97 ± 3%
With CsA (0.75 mM)	104 ± 0	40 ± 0	21 ± 1%	88 ± 1%
With CsA (1.00 mM)	105 ± 1	39 ± 5	23 ± 4%	74 ± 6%

## Data Availability

Data are contained within the article and Supplementary Materials.

## Supplementary Materials

The following supporting information can be downloaded at: https://www.mdpi.com/article/10.3390/pharmaceutics16040493/s1, Figure S1: Cytotoxicity studies showing the LC_50_ of the DOPE: DOTAP (1:1) after 24 h of incubation with the different liposome concentrations with the MDCK II SENS/ABCB1/ABCG2/ABCC1 cell lines; Figure S2: The concentration dependent effect of drug Free MFVs on the inhibitory potency of the CsA towards ABCB1. Lower concentrations of the MFVs is for the benefit of the inhibitory of the Hoechst 33342 binding site however at higher concentrations of the MFVs together with the high concentration of CsA leads to a disadvantage of the inhibitory effect of the CsA; Figure S3: The concentration dependent effect of drug Free MFVs on the inhibitory potency of the CsA towards ABCG2. Lower concentrations of the MFVs is for the benefit of the inhibitory of the Hoechst 33342 binding site however at higher concentrations of the MFVs together with the high concentration of CsA leads to a disadvantage of the inhibitory effect of the CsA; Figure S4: The concentration dependent effect of drug free MFVs on the inhibitory potency of the CsA towards ABCG2. MFVs have almost no effect on the inhibitory activity of the compound regarding the transport of the Pheophorbide A.
