# Supplementary material for: Membrane-Fusing Vehicles for Re-Sensitizing Transporter-Mediated Multiple-Drug Resistance in Cancer
Source: Pharmaceutics. 2024 Apr 2;16(4):493. doi: 10.3390/pharmaceutics16040493 (PMC11053612; doi:10.3390/pharmaceutics16040493)
Supplement: Supplementary file 1 [file pharmaceutics-16-00493-s001.zip › pharmaceutics-2872355-supplementary.pdf]

Supplementary:

Intrinsic toxicity of the MFVs. The toxicity of the drug free vehicles were determined to be LC_{50}=3.087 μM for cell line MDCK II Parental, μM, for MDCK II ABCB1 3.263 , and 3.073 μM for MDCK II ABCC1 and 3.175 μM for MDCK II BCRP. The toxicity of the DOPE: DOTAP vehicles showed no significant difference in the LC_{50} value between different cell lines with and without overexpression of ABC transporters. All cell biological assays were performed bellow these determined concentrations.

Log LC50	MDCK II ABCB1	MDCK II BCRP	MDCK II ABCC1	MDCK II Parental
	3.263	3.175	3.073	3.087

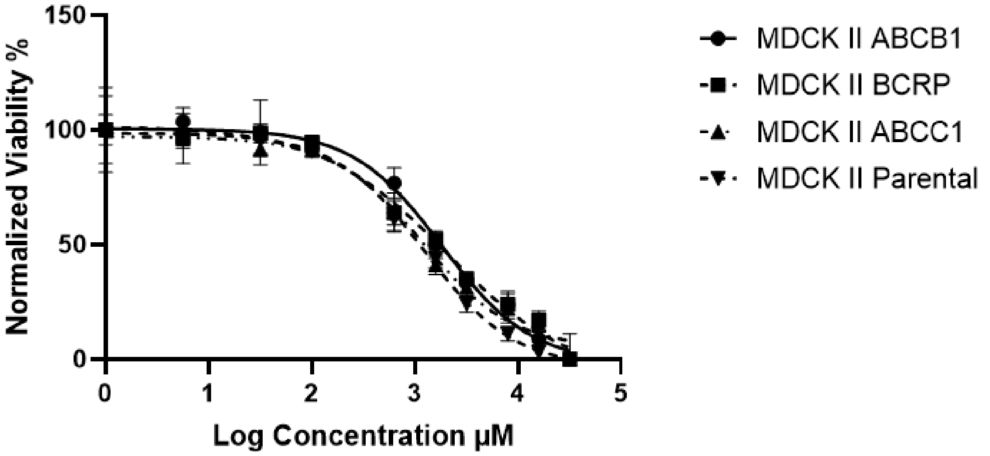

Supp. Figure S1 Cytotoxicity studies showing the LC_{50} of the DOPE: DOTAP (1:1) after 24 hours of incubation with the different liposome concentrations with the MDCK II SENS/ABCB1/ABCG2/ABCC1 cell lines

Concentration dependent effect of MFVs on the inhibitory effect of CsA:

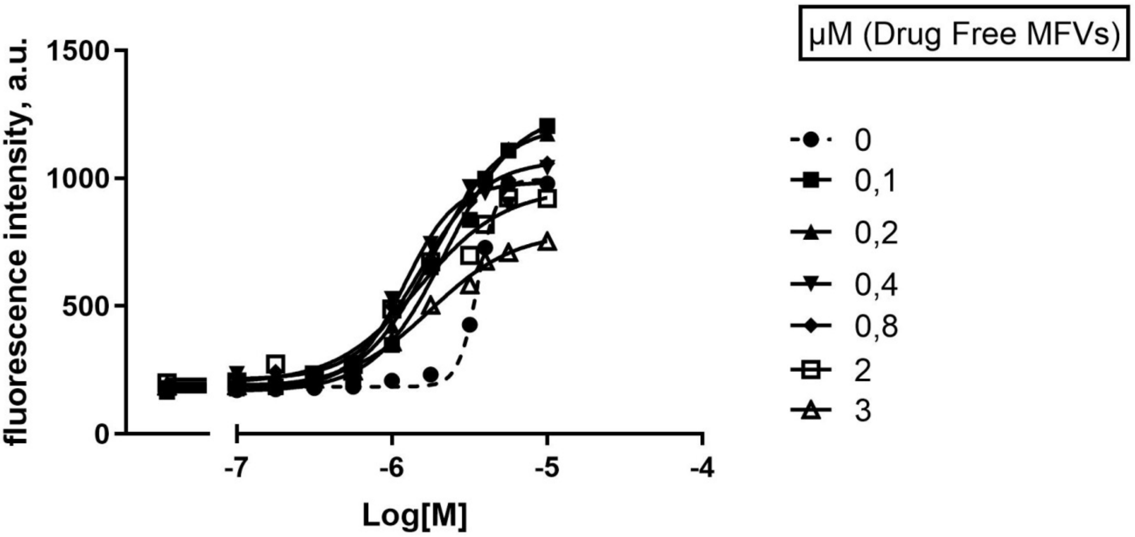

Supp. Figure S2 The concentration dependent effect of drug Free MFVs on the inhibitory potency of the CsA towards ABCB1. Lower concentrations of the MFVs is for the benefit of the inhibitory of the Hoechst 33342 binding site however at higher concentrations of the MFVs together with the high concentration of CsA leads to a disadvantage of the inhibitory effect of the CsA.

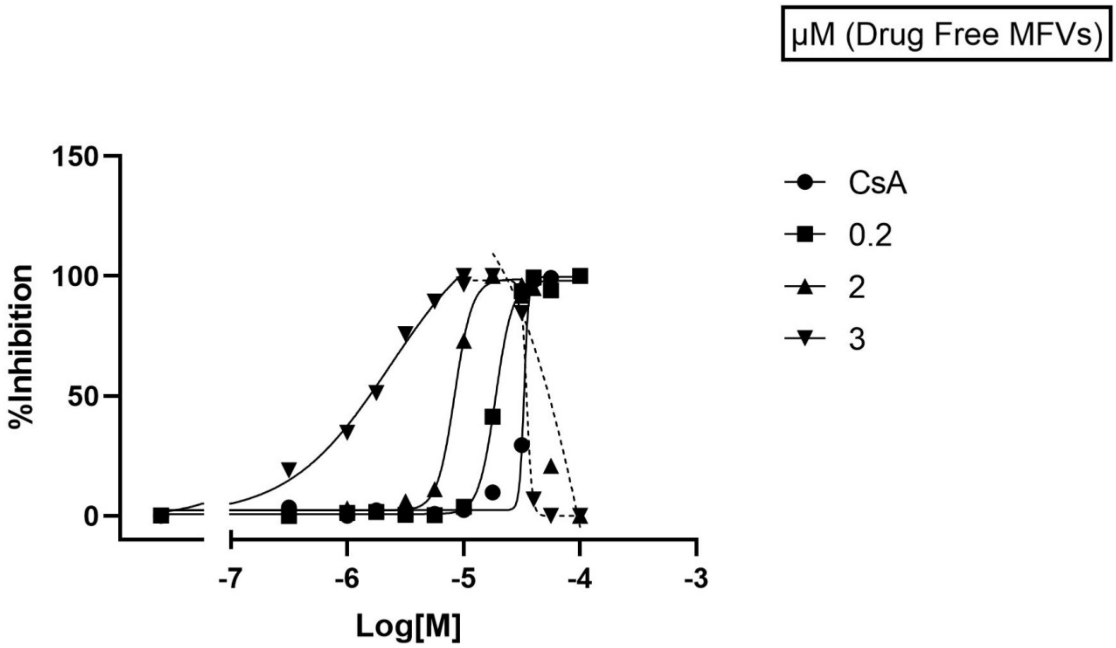

Supp. Figure S3 The concentration dependent effect of drug Free MFVs on the inhibitory potency of the CsA towards ABCG2. Lower concentrations of the MFVs is for the benefit of the inhibitory of the Hoechst 33342 binding site however at higher concentrations of the MFVs together with the high concentration of CsA leads to a disadvantage of the inhibitory effect of the CsA.

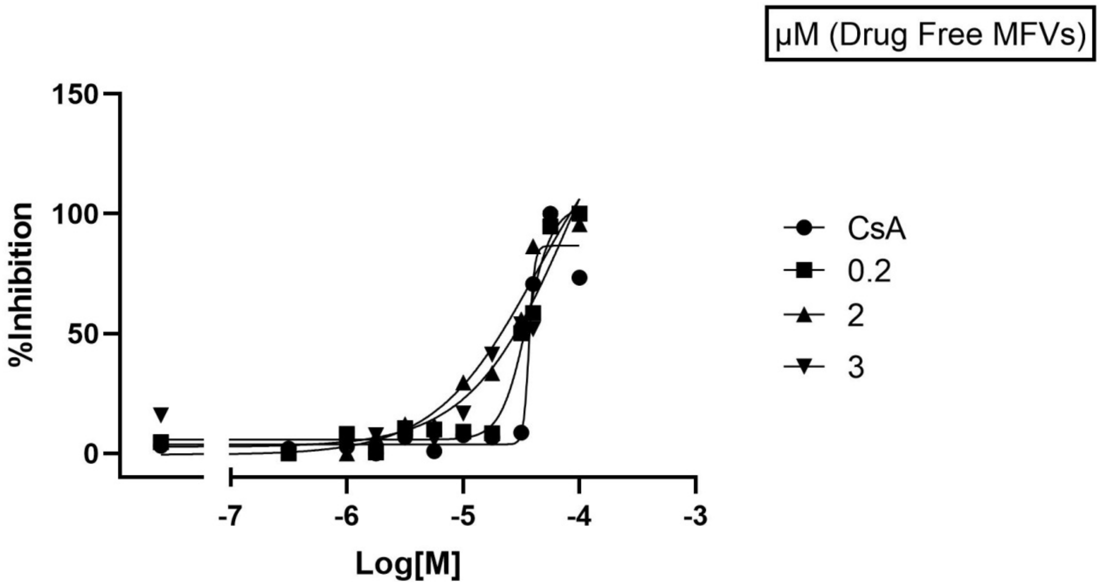

Supp. Figure S4 The concentration dependent effect of drug free MFVs on the inhibitory potency of the CsA towards ABCG2. MFVs have almost no effect on the inhibitory activity of the compound regarding the transport of the Pheophorbide A.

15
